# Chronology of Onset of Mental Disorders and Physical Diseases in Mental-Physical Comorbidity - A National Representative Survey of Adolescents

**DOI:** 10.1371/journal.pone.0165196

**Published:** 2016-10-21

**Authors:** Marion Tegethoff, Esther Stalujanis, Angelo Belardi, Gunther Meinlschmidt

**Affiliations:** 1 Division of Clinical Psychology and Psychiatry, Department of Psychology, University of Basel, Basel, Switzerland; 2 Division of Clinical Psychology and Epidemiology, Department of Psychology, University of Basel, Basel, Switzerland; 3 Faculty of Medicine, Ruhr-University Bochum, Bochum, Germany; Stellenbosch University, SOUTH AFRICA

## Abstract

**Background:**

The objective was to estimate temporal associations between mental disorders and physical diseases in adolescents with mental-physical comorbidities.

**Methods:**

This article bases upon weighted data (N = 6483) from the National Comorbidity Survey Adolescent Supplement (participant age: 13–18 years), a nationally representative United States cohort. Onset of Diagnostic and Statistical Manual of Mental Disorders, Fourth Edition lifetime mental disorders was assessed with the fully structured World Health Organization Composite International Diagnostic Interview, complemented by parent report. Onset of lifetime medical conditions and doctor-diagnosed diseases was assessed by self-report.

**Results:**

The most substantial temporal associations with onset of mental disorders preceding onset of physical diseases included those between affective disorders and arthritis (hazard ratio (HR) = 3.36, 95%-confidence interval (CI) = 1.95 to 5.77) and diseases of the digestive system (HR = 3.39, CI = 2.30 to 5.00), between anxiety disorders and skin diseases (HR = 1.53, CI = 1.21 to 1.94), and between substance use disorders and seasonal allergies (HR = 0.33, CI = 0.17 to 0.63). The most substantial temporal associations with physical diseases preceding mental disorders included those between heart diseases and anxiety disorders (HR = 1.89, CI = 1.41 to 2.52), epilepsy and eating disorders (HR = 6.27, CI = 1.58 to 24.96), and heart diseases and any mental disorder (HR = 1.39, CI = 1.11 to 1.74).

**Conclusions:**

Findings suggest that mental disorders are antecedent risk factors of certain physical diseases in early life, but also vice versa. Our results expand the relevance of mental disorders beyond mental to physical health care, and vice versa, supporting the concept of a more integrated mental-physical health care approach, and open new starting points for early disease prevention and better treatments, with relevance for various medical disciplines.

## Introduction

As the health of young people contributing to future population health and global economic development has been neglected yet, it has now become a ‘pressing issue’ [1]. The World Health Organization (WHO) and key medical journals such as the *Lancet* are dealing with the challenges that non-communicable diseases and mental disorders are imposing on the health care systems, and it has been claimed that these conditions need to be considered in global efforts in improvements of health, social policy, and health-care delivery [2–4].

The relevance of the integration of mental and physical health arises from adult studies documenting the systematic co-occurrence of mental disorders and physical diseases [3, 5–10]. Findings from longitudinal studies suggest that depression may be a risk factor for the development of cardiovascular diseases such as high blood pressure and coronary heart disease [11–13], autoimmune diseases such as type 1 diabetes, Crohn’s disease, and psoriasis [14], asthma, back pain, and migraines [12]. Temporal associations between depression and rheumatoid arthritis as well as respiratory diseases seem to be bidirectional [12, 15, 16]. Furthermore, posttraumatic stress disorder has been found to precede coronary heart disease [17], type II diabetes [18], and respiratory diseases [19], whereas irritable bowel syndrome may be an antecedent risk factor of epilepsy [20]. The healthcare significance of mental-physical comorbidity is underlined by diminished quality of life and unfavorable course of disease [21], substantial healthcare costs, higher treatment demand, longer treatment duration, and impaired treatment response in persons with mental-physical comorbidity [22, 23]. Integrating mental and physical health has gained attention and advanced into the focus of major journals, current strategic research goals and task forces [24–26].

Despite this relevance, the understanding of mental-physical comorbidity in children and adolescents is scarce, even though some studies support a relationship between mental disorders and physical diseases already during childhood or adolescence [27–35]. First evidence from longitudinal studies suggest that epilepsy may be a risk factor for the development of attention-deficit/hyperactivity disorder [36], that asthma may precede affective and anxiety disorders [37, 38], and that eating disorders may be an antecedent risk factor of a variety of physical diseases [31]. These studies, however, mostly used clinical samples and focused on selected mental or physical problems, and it has been suggested to further develop the life course perspective [39].

The current understanding of the etiology of mental-physical comorbidity is largely based on theoretical models attempting to explain how mental disorders and physical diseases come to be comorbid. These theories suppose that one condition operates as risk factor for the other, or that shared risk factors underlie both mental disorders and physical diseases [5, 40]. However, studies providing implications regarding trajectories in the development of mental-physical comorbidity are lacking. Therefore, knowledge on the temporal course of conditions has been claimed as highly relevant [41, 42].

To better understand the developmental trajectories of mental-physical comorbidity, the main objective of this study was to estimate in adolescents with mental-physical comorbidity the temporal association of mental disorders and physical diseases, using data on the age of onset of a wide range of mental and physical morbidities from a representative national cohort study.

## Methods

### Study sample

We based this study on data of the National Comorbidity Survey Replication Adolescent Supplement (NCS-A), a national representative survey of initially 10148 United States (US) adolescents (ages 13–18), of which 10123 were students at the time of the survey. Data collection took place between February 2001 and January 2004 [43–45]. Further details on the study protocol of the NCS-A have been described previously [34, 43, 44, 46]. We based our analyses on a subsample of 6483 NCS-A participants for which parents or guardians completed a Self Administered Questionnaire (SAQ), as described previously [34]. Details on the subsample for which no parent report was available, and differences between these two subsamples, are available in supplementary Table 1 of a previous publication that was based on the same dataset [35]. Adolescent and parent provided written informed consent, and the study protocol was approved by the Human Subjects Committee of Harvard Medical School and the University of Michigan.

**Table 1:** Sociodemographic characteristics of the study sample* (*N* = 6483).

Sociodemographic factor	Category	*n*	Weighted %
**Sex**	Female	3333	48.76
	Male	3150	51.24
**Age**	13–14 y	2611	35.92
	15–16 y	2528	41.88
	17–18 y	1344	22.20
**Race**	Hispanic	758	14.38
	Afro-American	1097	15.07
	Other	371	4.99
	Caucasian	4257	65.55
**Parental education (highest level of either parent)**	Less than high school	746	12.32
	High school	1852	29.33
	Some college	1364	21.31
	College grad	2521	37.04
Poverty index ratio**	≤1.5 (poor)	925	14.59
	≤3	1218	19.26
	≤6	2139	32.65
	>6	2201	33.51
**Region**	Northeast	1273	18.15
	Midwest	2081	23.27
	South	2100	36.02
	West	1029	22.56
**Urbanicity†**	Metropolitan area	2645	45.68
	Other urban area	2242	39.48
	Rural area	1596	14.83
**Number of biological parents living with the adolescent**	0	528	8.86
	1	2284	36.46
	2	3671	54.68
**Birth order**	Oldest	2314	38.81
	Youngest	1947	28.42
	Others	2222	32.77
**Number of siblings**	0	323	5.24
	1	1853	29.30
	2	1745	27.44
	3 or more	2562	38.02

Abbreviations: y, years

*Subsample of the National Comorbidity Survey-Adolescent Supplement (NCS-A) including all participants providing self- and parent-reported information on mental disorders.

**Poverty index ratio: The ratio of family income to the poverty threshold of the family, for which the poverty threshold depends on family size [62].

†Urbanicity was categorized based on the classification criteria of the US Census Bureau of 2000: ‘Metropolitan’ corresponds to 1000 or more people per square mile, ‘Other urban area’ corresponds to at least 500 people per square mile, ‘Rural area’ corresponds to other regions [64].

### Diagnostic Assessment

#### Mental disorders

To assess lifetime mental disorders, trained interviewers administered a computer-assisted version of the WHO Composite International Diagnostic Interview (CIDI) Version 3.0 [45, 46]; details have been described previously [43, 47]. Additional information on adolescents’ mental health was collected from parents or guardians based on the SAQ focusing on attention-deficit/hyperactivity disorder, conduct disorder, oppositional defiant disorder, major depressive disorder, and dysthymic disorder, because collecting information from parents about those disorders has been found to be diagnostically valuable [48–50]. Information from adolescents and parents was combined. A mental disorder was considered present when diagnostic criteria were met either based on information obtained from adolescent or parent, and, in case of discrepancies, the earlier age was used as age of onset.

We grouped specific mental disorders into the following disorder categories: Any affective disorder (major depressive disorder, dysthymia, and bipolar I or II disorder), any anxiety disorder (agoraphobia, generalized anxiety disorder, social phobia, specific phobia, panic disorder, posttraumatic stress disorder, and separation anxiety disorder), any behavior disorder (attention deficit hyperactivity disorder, oppositional defiant disorder, and conduct disorder), any substance use disorder (alcohol abuse or dependency and drug abuse or dependency), any eating disorder (anorexia nervosa, bulimia nervosa, and binge eating disorder). If an adolescent, based on either adolescent or parent report, fulfilled diagnostic criteria for more than one disorder with a certain disorder category, we used the earliest age as age of onset of the respective disorder category.

#### Physical diseases

The lifetime presence (‘yes’, ‘no’) and the age of onset of physical diseases were assessed solely with adolescent self-report, based on a checklist on chronic conditions, which has been applied in the US National Health Interview Survey in similar form [51]. Checklists have been extensively used in national studies [51–54]. It has been shown that children are able to reliably and validly report on their health already at early life stages [55–57]. In this respect, self-report and medical records show good concordance [58], with checklists being superior to data obtained from routine data sources in terms of completeness and accuracy [59].

**Fig 1:**
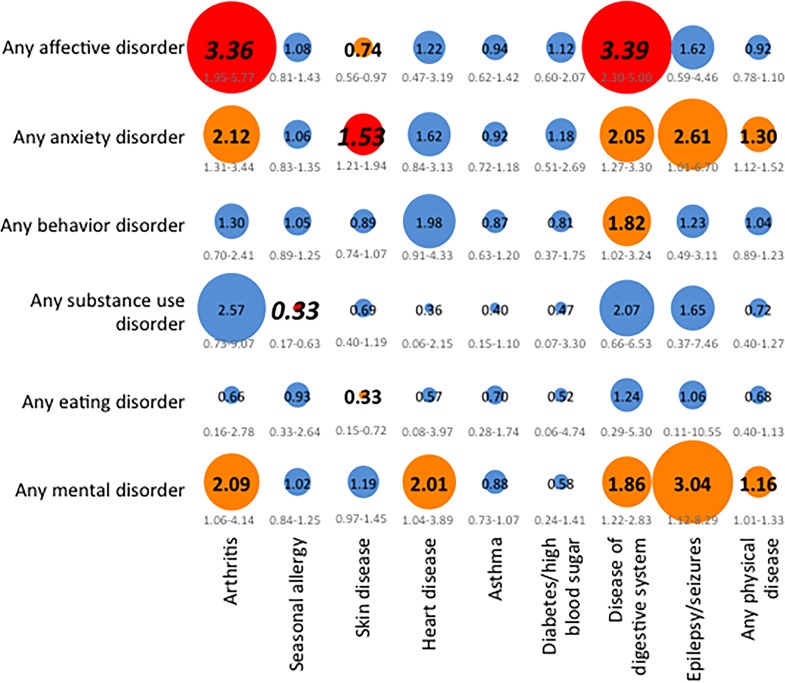
Adjusted discrete-time proportional hazard models estimating the temporal associations of mental disorders predicting subsequent physical diseases. Note: We based our analyses on completer sample sizes* of the total study sample (N = 6483), and adjusted for sociodemographic variables shown in Table 1. The strength of the associations (hazard ratios (HR) is illustrated by the circle diameter, given in the circles, and 95% confidence intervals, given below the circles). Blue color of the circle (and HRs provided in small standard type font) represent p≥0.05 in the total study sample; orange color of the circle and HRs provided in medium-sized bold type font represent p<0.05 in the total study sample and in less than two independent subsamples; red color of the circle and HRs provided in large bold and italic type font represent p<0.05 in the total study sample and in at least two independent subsamples. * Due to missing information on physical diseases from adolescent self-report, sizes of the completer samples are as follows: arthritis: n = 6473, seasonal allergy: n = 6475, skin disease: n = 6479, heart disease: n = 6481, asthma: n = 6477, diabetes/high blood sugar: n = 6481, disease of the digestive system: n = 6481, epilepsy or seizures: n = 6481, any physical disease: n = 6469.

**Fig 2:**
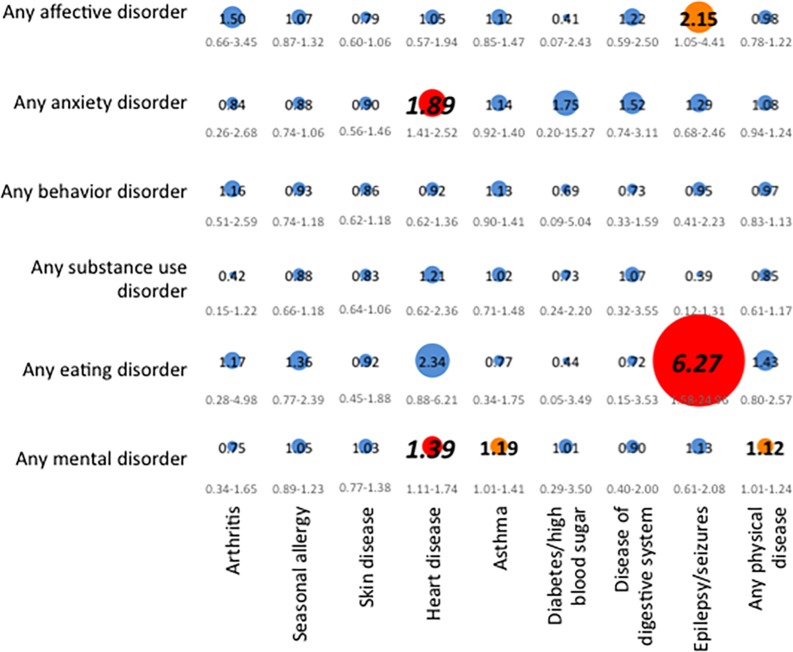
Adjusted discrete-time proportional hazard models estimating the temporal associations of physical diseases predicting subsequent mental disorders. Note: We based our analyses on completer sample sizes* of the total study sample (N = 6483), and adjusted for sociodemographic variables shown in Table 1. The strength of the associations (hazard ratios (HR) is illustrated by the circle diameter, given in the circles, with 95% confidence intervals, given below the circles). Blue color of the circle (and HRs provided in small standard type font) represent p≥0.05 in the total study sample; orange color of the circle and HRs provided in medium-sized bold type font represent p<0.05 in the total study sample and in less than two independent subsamples; red color of the circle and HRs provided in large bold and italic type font represent p<0.05 in the total study sample and in at least two independent subsamples. * Due to missing information on physical diseases from adolescent self-report, sizes of the completer samples are as follows: arthritis: n = 6473, seasonal allergy: n = 6475, skin disease: n = 6479, heart disease: n = 6481, asthma: n = 6477, diabetes/high blood sugar: n = 6481, disease of the digestive system: n = 6481, epilepsy or seizures: n = 6481, any physical disease: n = 6469.

Physical diseases included in our study can be seen in Figs 1 and 2, and further details have been described previously [34].

### Statistical analyses

We used weighted data in all statistical analyses, which were conducted with STATA/MP 11 (Stata Corporation, College Station, Texas). Weights were provided with the NCS-A dataset, and had been calculated based on a wide range of sociodemographic variables with regard to [43, 44] to ensure representativeness of the NCS-A study sample with the US adolescent population. We estimated temporal relationships between mental disorders and physical diseases by calculating separate discrete-time proportional hazard models with a non-parametric baseline hazard function using complementary log-log regression, with one of the major classes of mental disorders or ‘any mental disorder’ and one physical disease or ‘any physical disease’ defined as outcome and as time-varying predictor, respectively, and vice versa [60]. We present hazard ratios and their 95% confidence intervals. If diagnostic criteria for more than one mental disorder were fulfilled within a mental disorder class, we used the age of onset of the first mental disorder in this class as age of onset of the total class. As we had to deal with complex survey data, we applied the Taylor series linearization method. In accordance with previous studies [61, 62], we included sociodemographic variables shown in Table 1 in our analyses to control for potential confounding. Adjusted results are presented. To account for the large number of pairwise test, we used an internal subsampling strategy, as previously described [34, 63].

For a low number of subjects information on physical diseases from adolescent self-report was missing. We restricted each analysis to subjects with complete data (see Figs 1 and 2 for completer sample sizes according to each physical disease category). We defined statistical significance at 0.05 and two-sided tests were applied.

## Results

### Study cohort descriptives

Table 1 summarizes the study cohort’s sociodemographic characteristics (N = 6483).

### Temporal prediction of physical diseases by mental disorders

Results of the adjusted discrete-time proportional hazard models estimating the temporal associations between physical diseases and mental disorders, with mental disorders preceding physical diseases, in the total sample are presented in Fig 1 (results from subsamples available on request). The most substantial associations included those of affective disorders with arthritis and diseases of the digestive system, between anxiety disorders and skin diseases, and between substance use disorders and seasonal allergies. In support of transparency, results of the crude regression models are presented in S1 Table.

### Temporal prediction of mental disorders by physical diseases

2 presents results of the adjusted discrete-time proportional hazard models of the associations between mental disorder classes and physical diseases, with physical diseases preceding mental disorders, in the total sample (results from subsamples available on request). The most substantial associations included those of heart diseases with any mental disorder and anxiety disorders, and between epilepsy and eating disorders. In support of transparency, results of the crude regression models are presented in S2 Table.

We provide information on age of onset intervals between the temporal relations of our most robust findings in S3 Table.

## Discussion

This article provides temporal association estimates of lifetime mental disorders and physical diseases, based on data from 6483 adolescents of a nationally representative cohort. The most substantial results indicate that affective disorders are a risk factor of arthritis and diseases of the digestive system, that anxiety disorders are a risk factor of skin diseases, and that substance use disorders are a protective factor of seasonal allergies. Vice versa, heart diseases may indicate a risk of anxiety disorders and any mental disorder, and epilepsy a risk of eating disorders.

Our results contribute to previous findings on mental-physical comorbidity mostly resulting from association studies in clinical or population-based samples in adults and documenting comprising relationships between mental disorders and physical diseases [3, 5, 6], including the comorbidity patterns observed in the present study [65–70]. However, as yet, there has been no evidence suggesting a link between substance use disorders and allergies [71], and even though comorbidity between epilepsy and mental disorders has been described in children [28, 30], epidemiological data on the co-occurrence of epilepsy and eating disorders are lacking.

There is rare evidence from adult intervention trials providing insight into the developmental trajectories of co-occurring mental disorders and physical diseases. A contribution of depression in arthritis is supported by a study demonstrating benefits of improved depression care that extended beyond reduced depressive symptoms and included decreased pain in older adults with arthritis and comorbid depression [72]. That anxiety may precede the onset of skin diseases is elucidated by studies of patients with atopic dermatitis reporting improvement in anxiety levels and skin conditions after psychotherapy [73, 74]. For eating disorders and epilepsy, it has been hypothesized based on case reports, that epilepsy arising from a right hemispheric focus and right frontal intracerebral lesions–with their close relationship to the limbic system–could play a role in the development of eating disorders [75, 76]. This view is supported by the emerging importance of antiepileptic drugs in the treatment of eating disorders [77]. Finally, for affective disorders preceding diseases of the digestive system, our findings are in line with positive associations between current depression and subsequent disease activity in adult patients with Crohn’s disease or the development of ulcers in previously ulcer-free subjects [78, 79].

In contrast to findings from meta-analyses of studies in adults [80, 81], our data do not suggest anxiety as a risk factor of heart diseases, which may be due to the young age of subjects, as anxiety-induced pathophysiological processes might take decades to develop. Vice versa, the prognostic relevance of cardiovascular diseases for anxiety disorders is less clear in the adult literature. Even though there is some evidence for increased anxiety levels after myocardial infarction [82–84], prospective data providing pre-infarction information is mostly not available, and studies addressing causality are lacking.

Different biological, behavioral, cognitive, and social pathways mediating the relationships between mental disorders and physical diseases have been proposed, but even though study designs to inform about developmental trajectories have already been applied successfully [85], specific comorbidity patterns remain to be determined [5, 40, 86]. Until then, the available data may help to generate hypotheses on the nature of these pathways.

With regard to depression and arthritis, previous work documents the pain-enhancing potential of brain circuits that may be disturbed in depression [87] and, vice versa, the analgesic effects of antidepressants [88], indicating that depression-related brain networks might contribute to the etiology of arthritis. Further pathway candidates are the immune system and the hypothalamic-pituitary-adrenal (HPA) axis, as local inflammation, followed by a systemic reaction, and inappropriately low secretion of cortisol are typical features of arthritis [89, 90], and disturbances of the immune system and the HPA axis have been described in persons with depression [91, 92].

Regarding depression-related onset of diseases of the digestive system, the pathophysiology of the brain-gut axis, involving the corticotropin-releasing factor system [93] may play a role [94], as experimental and clinical studies have demonstrated that acute and chronic stress have impacts on the gastrointestinal system, being permissive in the development of gut diseases [95].

In terms of potential mechanisms underlying the observed prediction of skin diseases by anxiety disorders, it is of note that psychological stress has not only been associated with atopic dermatitis symptom severity [96], but also with various skin health-relevant immune alterations, including slowed wound healing and augmented induction of inflammatory processes and immunoglobulin E (IgE) production [97, 98]. To date, there is only preliminary evidence on the psychoneuroimmunology of anxiety disorders, suggesting that high levels of anxiety might be associated with impaired cellular immunity and IgE synthesis [99, 100].

The reduced risk of seasonal allergy related to substance use disorders in our study may be a consequence of increased consumption of certain substances, for example alcohol, and related immunological changes [101–103], but such positive consequences should be interpreted with caution as it is well established that substance use disorders are associated with increased risk of morbidity and mortality [104].

Body perception and interoceptive conditioning may contribute to the heart disease-related increased risk of anxiety disorders [105]: Heartbeat sensitivity has been shown to be increased in persons suffering from congenital heart diseases compared to healthy controls [106] and studies using heartbeat perception tasks in anxiety disorders support the notion of higher interoceptive sensitivity towards the heartbeat as etiological factor in anxiety diseases [107]. Changes in neuronal structure and function resulting from epileptic seizures [108], possibly contribute to the risk of eating disorders related with epilepsy. A systematic review of case reports concluded that although simple changes in appetite and eating behavior occurred with hypothalamic and brain stem lesions, the characteristic psychopathology of eating disorders was associated with right frontal and temporal lobe damage [109]. On a molecular level, it has been documented that 5-hydroxytryptamine (serotonin) receptor 2C, G protein-coupled (HTR2C)-receptor-deficient mice showed disturbed feeding behavior and were prone to spontaneous death from seizures, suggesting that 5-HT2C receptors may play a role in linking eating disorders and epilepsy [110].

Strengths of our study include the large nationally representative sample [43], the broadness of mental disorders and physical diseases included, the use of a fully structured diagnostic interview for the assessment of mental disorders, with good quality criteria [43, 47], and the integration of child and parent information [50]. Good response rate and the minimal amount of missing data make it unlikely that loss of subjects has introduced selection bias. Still, the results of this study should be interpreted in light of several limitations; some have been discussed previously, including self-report measures of physical diseases [34], the cross-sectional design, and the use of retrospective data, involving the risk of recall bias [46, 111]. Specifically, the wording of the questions in the physical diseases checklist in the CIDI (*"Did a doctor or other health professional ever tell you that you had any of the following illnesses*…*"*) might have led to underestimated values, because to positively answer any of these questions the adolescent must have sought a health professional and recalled the diagnosis.

However, a suitable longitudinal dataset allowing studying the chronology of onset of mental disorders and physical diseases in mental-physical comorbidity patterns is lacking while needed to corroborate our findings. Until then, these findings are important to guide future research by providing hypotheses, not least given the novel probing strategy of the National Comorbidity Surveys that has been shown to increase the accuracy of age of onset reports [112]. Moreover, the young and relatively homogenous age range of NCS-A participants within the peak-onset period of mental disorders [113] diminishes the risk of potential bias by age-related impairment in the recall of age of illness onset [114]. Furthermore, lifetime prevalence estimates (reported by our group for the main categories of mental disorders and for physical diseases in [34] and in [62] for specific mental disorders) and age of onset distributions of mental disorders and physical diseases (see S4 Table) are generally in line with previous findings [115–134]. Still, participants could have been rather young at disease onset that could have occurred a decade or more prior to the assessment. This might have introduced recall bias. However, as already mentioned, previous work demonstrated that children’s self-reports on their health are largely reliable and valid [55–57].

Moreover, according to the risk-factor concept by Kraemer and colleagues [135] and due to the cross-sectional design of the study, the presented findings cannot inform about ‘causal risk factors’ but rather about ‘risk factors’ defined as factors preceding the outcome. Besides the temporal relationship, other aspects suggesting causality [136] may be considered, including the strengths of the relationships, for example those between affective disorders and arthritis, with HRs > 3, the specificity of associations, their mechanistic plausibility as discussed above, as well as evidence from the few intervention trials or consistency with the few prospective studies depicted above. Finally, we restricted our analyses to the main categories of mental disorders and physical diseases instead of focusing on subcategories. This hampered integration of results into the literature but ensured sufficient number of cases for each comorbidity pattern, thereby complying with statistical assumptions.

Given the high lifetime prevalence of some comorbidity patterns [34, 35], the partly substantial temporal relationships between lifetime mental disorders and physical diseases, and the high burden for the individual and health economics [21–23, 66], our findings carry relevance for psychiatric and medical health care and the roles of psychiatrists and other medical specialists in patient management [26], and they can inform research priorities and guide task forces, health policy plans and medical education [137]. In line with current strategic research goals [24, 25, 138], our results may pave the way to improve diagnostic approaches, prevention and treatment of mental-physical comorbidity, for example by considering that treatment of a mental disorder may have implications for a physical disease, and vice versa [139].

A large body of evidence from the WHO World Mental Health Survey documented that the epidemiology of mental-physical comorbidity in adults is comparable worldwide [5], suggesting that the temporal course of onset of mental disorders and physical diseases in adolescents might as well be similar worldwide. However, generalizability of our findings from an adolescent sample on an adult population might be limited, for instance, due to the increasing influence of lifestyle-related factors across the lifespan and, hence, the rather late onset of certain physical disorders [140–142].

Future studies should, besides surveying longitudinal data, include subclinical manifestations of mental disorders and physical diseases, for example using non-invasive measures of arterial structure and function for heart diseases, that occur before the onset of symptoms, in order to better understand the temporal sequence of the relationships. Additionally, it may be worth scrutinizing the comorbidity of mental and physical conditions with regard to the relevance of age of onset, duration of the conditions, and temporal distance between ages of onset (in future studies). Moreover, randomized-controlled intervention trials in representative populations and animal models would be important to shed light on the causality and underlying biological, psychological and behavioral mechanisms of the relationships between mental disorders and physical diseases that we revealed, to foster the development of interdisciplinary preventive approaches and interventions, including the development of clinical guidelines dealing simultaneously with mental disorders and physical diseases [143].

To the best of our knowledge, this is the first comprehensive study of the temporal association of mental disorders and physical diseases in adolescents with mental-physical comorbidity in a nationally representative survey, based on data from 6483 subjects. The most substantial results indicate that affective disorders may increase the risk of arthritis and diseases of the digestive system, that anxiety disorders may increase the risk of skin diseases, and that substance use disorders may decrease the risk of seasonal allergies. Vice versa, heart diseases may indicate a risk of anxiety disorders and any mental disorder, and epilepsy a risk of eating disorders. The clear temporal relationships between mental disorders and physical diseases for specific comorbidity patterns suggest that certain mental disorders may be risk factors of certain physical diseases at early life stages, and vice versa. These results predominantly expand the relevance of mental disorders in adolescence beyond mental health care to physical health care, and vice versa, supporting the concept of integrative care, and open new starting-points for early disease prevention and better treatments, which is relevant for various medical disciplines.

## Supporting Information

S1 Table Discrete-time proportional hazard models for lifetime mental disorders (time-varying) predicting physical diseases (crude models). (XLS)

S2 Table Discrete-time proportional hazard models for lifetime physical diseases (time-varying) predicting mental disorders (crude models). (XLS)

S3 Table Age of onset intervals of temporal associations between mental disorders preceding physical diseases and vice versa in participants reporting both conditions. (XLS)

S4 Table Ages of onset by physical disease/mental disorder category. (XLS)
